# New grading system for upper urinary tract dilation using magnetic resonance urography in patients with Neurogenic Bladder

**DOI:** 10.1186/1471-2490-14-38

**Published:** 2014-05-22

**Authors:** Limin Liao, Fan Zhang, Guoqing Chen

**Affiliations:** 1 Department of Urology, China Rehabilitation Research Center, 10 Jiaomen Beilu, Beijing 100068 Fentai District, China; 2 Department of Urology of Capital Medical University, 10 Youanmenwai Xitoutiao, Beijing 100069 Fentai District, China

**Keywords:** Upper urinary tract dilation, Grade, Hydronephrosis, Ureteral dilation, Magnetic resonance urography, Neurogenic bladder

## Abstract

**Background:**

In patients with neurogenic bladder (NB), elevated intravesical pressures can be transmitted to the upper urinary tract, causing hydronephrosis (HN) and ureteral dilation (UD), which are referred to as upper urinary tract dilation (UUTD). Ureteral obstruction at the bladder wall is another cause for UUTD, but is less of a concern. UUTD can lead to chronic renal failure. Therefore, evaluation and protection of UUT function is extremely important in the management for NB. Currently, the most common method by which to detect HN and UD is ultrasonography (US). The Society for Fetal Urology (SFU) established an US HN grading system in 1993, but this system was found to have some defects. The purpose of this study is to describe a new grading system for UUTD, including both HN and UD, based on magnetic resonance urography (MRU) and to correlate the new grading system with the SFU grading system for HN.

**Methods:**

A retrospective review of 70 patients with unilateral or bilateral UUTD was completed. Ninety-five sides in patients with UUTD were graded by the MRU-UUTD and SFU-HN grading systems. The results from the two grading systems were compared for each UUTD.

**Results:**

The MRU-UUTD grading system revealed the following percentages for each grade: grade 0, 0; 1, 10.5%; 2, 19%; 3, 42.1%; and 4, 28.4%. The SFU-HN grading system revealed the following percentages for each grade: 0, 0; 1, 10.5%; 2, 19%; 3, 36.8%; and 4, 33.7%. There was no significant systematic difference between the two grading systems (*p* > 0.05), but a significant difference between grades 3 and 4 (*p* < 0.05).

**Conclusions:**

The MRU-UUTD grading system correlates well with SFU-HN grade, provides an objective and comprehensive evaluation for UUTD, and can be used for longitudinal monitoring of UUTD. This new grading system allows for better informed clinical decision-making, identifying changes in UUTD.

## Background

Neurogenic bladder (NB) refers to bladder and/or urethral dysfunction caused by various nervous system diseases. The presence of high bladder pressure is one of the devastating problems for patients with NB. Elevated intravesical pressures can be transmitted to the upper urinary tract (UUT), causing hydronephrosis (HN) and ureteral dilation (UD), which are referred to as upper urinary tract dilation (UUTD). Ureteral obstruction at the bladder wall is another cause for UUTD, but is less of a concern. UUT dilation or deterioration can lead to chronic renal failure. Therefore, evaluation and protection of UUT function is extremely important in the management for NB [1,2]. Currently, the most common method by which to detect HN and UD is ultrasonography (US). HN has been subjectively classified as mild, moderate, and severe. The Society for Fetal Urology (SFU) established an US grading system based on renal sinus splitting patterns and dilation of the renal pelvis and calyces in 1993 [3]. Although the SFU grading system has been widely accepted by the US specialists, the SFU grading system is not popular amongst clinical urologists. Moreover, deficiencies in the SFU grading system have been identified, especially with respect to the differentiation of severe (grades III and IV) HN [4,5]. Other investigators have suggested improvements or complementary approaches to the SFU grading system based on US [4-7]; however, none of the improvements or complementary approaches has gained widespread use. Moreover, US findings are not easy to interpret by a clinical urologist (particularly prominent in China) because the kidneys and ureters are not shown in the same image, and is thus a somewhat subjective assessment for UUTD. In the present study we describe a new grading system for UUTD, including HN and UD, based on magnetic resonance urography (MRU), correlate this new grading system with the SFU grading system, and attempt to provide clinicians with a more objective, intuitive, and understandable new method for UUTD grading.

## Methods

The current study was a retrospectively observational study approved by the Ethics Committee of the China Rehabilitation Research Center. The patient records were anonymized and de-identified prior to analysis. A retrospective review of 120 patients who were diagnosed with NB and underwent augmentation enterocystoplasty (AE) at our center between 2005 and 2013 was completed. NB resulted from traumatic spinal cord injury (SCI), spina bifida, and other causes (e.g., tumors). All of the patients underwent the following pre-operative routine examinations: US of the urinary tract; MRU; isotope renography; and video-urodynamics. The same US examiner completed all US examinations of UUT and reports of HN grades according to the SFU system. All MRU images were reviewed by an urologist. The patients with vesicoureteral reflux (VUR), but without UUTD, were excluded from the 120 patients; thus, 70 patients with unilateral or bilateral UUTD (48 males and 22 females; mean age, 25 years) were included in this study. The UUTD from 70 patients was graded via a developed grading system that was previously reported in Chinese and very briefly described in English by Liao [8,9]. This new UUTD grading system based on MRU findings was systematically described in detail in English, and corresponds to the SFU and Onen grading systems based on US findings [3,4], but there are some modifications, including the use of objective MRU findings and renal parenchyma changes in grade 3 and 4 definitions. The MRU-UUTD grading system is comprehensively described from the MRU coronal and transverse image panel, and the maximum intensity projection MRU:

**Figure 1:**
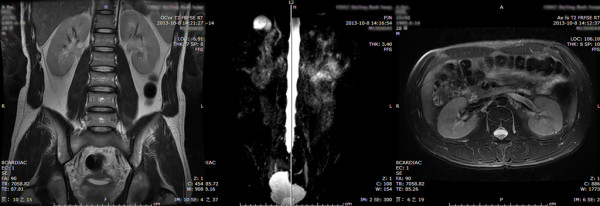
Zero grade UUTD (bilateral sides): the coronal and transverse MRU show that the central renal complex is closely apposed without any separation; the maximum intensity projection MRU does not show the visualized renal pelvis and ureters.

**Figure 2:**
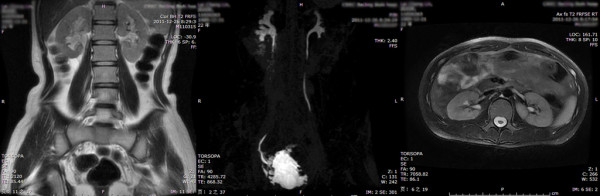
**First grade UUTD (bilateral sides): the coronal and transverse MRU reveal a slight separation of the central renal complex, but there are no visualized calices.** The maximum intensity projection MRU shows the visualized renal pelvis and ureters, but the ureter is < 7 mm.

**Figure 3:**
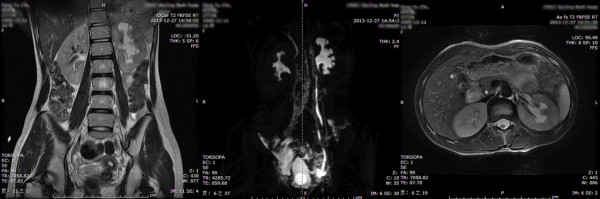
**Second grade UUTD (left side): the coronal and transverse MRU show that the renal pelvis is dilated; one or more calices may be visualized, but the renal parenchyma over the calices is normal.** The maximum intensity projection MRU shows the dilated renal pelvis and ureters, but the ureter is < 10 mm.

**Figure 4:**
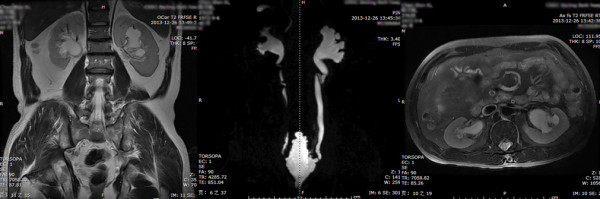
**Third grade UUTD (left side): the coronal and transverse MRU show that the renal pelvis is further dilated and there are fluid-filled calices throughout the kidney; the renal parenchyma over the calices is beginning to thin, but renal parenchyma loss is < 50%.** The maximum intensity projection MRU shows the dilated renal pelvis and ureters, the ureter is tortuous, and < 15 mm.

**Figure 5:**
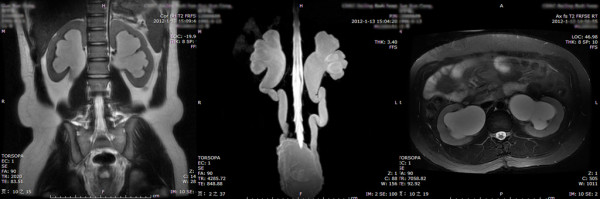
**Fourth grade UUTD (bilateral sides): the coronal and transverse MRU show that the bilateral renal pelvises are severely dilated, there are fluid-filled calices throughout the kidneys, and the renal parenchyma is significantly thinned (renal parenchyma loss > 50%).** The maximum intensity projection MRU shows the severely dilated renal pelvis and ureters; the ureter is significantly tortuous and > 15 mm.

1. Grade 0: The central renal complex is closely apposed without any separation and the ureteral dilation from the coronal and transverse MRU images and the maximum intensity projection MRU (Figure 1).2. Grade 1: There is slight separation of the central renal complex, but no visualized calices; the ureter is < 7 mm from the coronal and transverse MRU images and the maximum intensity projection MRU (Figure 2).3. Grade 2: The renal pelvis is dilated, one or more calices may be visualized, but the renal parenchyma over the calices is normal; the ureter is < 10 mm from the coronal and transverse MRU images and the maximum intensity projection MRU (Figure 3).4. Grade 3: The renal pelvis is further dilated and there are fluid-filled calices throughout the kidney. The renal parenchyma over the calices is beginning to thin, but the renal parenchyma loss is < 50%. The ureter is tortuous and < 15 mm from the coronal and transverse MRU images and the maximum intensity projection MRU (Figure 4).5. Grade 4: As in grade 3, but the renal parenchyma is significantly thinned (renal parenchyma loss > 50%). The ureter is severely tortuous and > 15 mm from the coronal and transverse MRU images and the maximum intensity projection MRU (Figure 5).

The HN grades from US and the UUTD grades from MRU were compared for each patient. The results were reported as the percentage of each grade on US and the MRU grading systems. Statistical analysis was performed using SPSS (11.5.0; SPSS, Inc., Chicago, IL, USA). The *Ridit* analysis was used for comparisons, and statistical significance was set at a *p* < 0.05.

## Results

Seventy patients had a total of 95 sides with UUTD (including HN and UD); the number of patients with unilateral and bilateral UUTD was 45 and 25, respectively. The percentage of HN grades based on US and the UUTD grades based on MRU are shown in Table 1.

**Table 1:** Comparison of percentage of US-HN and MRU-UUTD grades in 95 sides in patients with UUTD

	**Grade 0 No (%)**	**Grade 1 No (%)**	**Grade 2 No (%)**	**Grade 3 No (%)**	**Grade 4 No (%)**
MRU-UUTD grading system	0 (0)	10 (10.5)	18 (19)	40 (42.1)*	27 (28.4)*
US-HN grading system	0 (0)	10 (10.5)	18 (19)	35 (36.8)	32 (33.7)

*means: *p* < 0.05.

**Figure 6:**
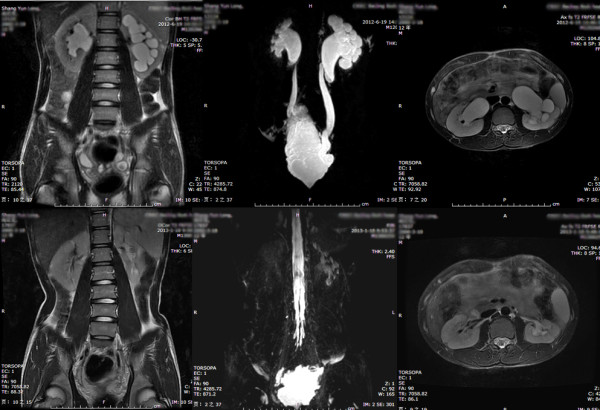
Improvement of UUTD grades by MRU after 6 months of AE: pre-operative UUTD grades of the right and left kidney were 3 and 4, respectively(the upper part), post-operative UUTD grades of the right and left kidney became 0 and 1, respectively (the lower part), and a significant improvement is shown.

Table 1 shows that there was no significant difference in the distribution of the grades between the MRU-UUTD and US-HN grading systems (*p* > 0.05), but there was a significant difference in grades 3 and 4 between the two systems (*p* < 0.05). The MRU-UUTD grade correlated well with the SFU-HN grade. With respect to the US-HN grade according to the SFU grading system, 5.3% of patients with UUTD were changed from grade 4 to grade 3 according to the MRU-UUTD grading system.The MRU-UUTD grade can be used for longitudinal monitoring of HN and UD, including pre- and post-operative observation; an example of evaluation for the outcome of the AE operation is shown in Figure 6.

## Discussion

The protection of UUT function is an extremely important goal in the management of NB patients. The evaluation of UUT function is the first step in achieving this goal. HN is most commonly classified as mild (5.0-9.9 mm), moderate (10.0-14.9 mm), or severe (≥15.0 mm) by renal US. Currently, the main paradigm for describing and grading HN is the SFU grading system, which is based on US findings. The appearance of the calyces, dilation of the renal pelvis, and thinning of the renal parenchyma are key in determining the SFU-HN grade [3]. Indeed, it is known that HN and renal parenchymal thickness have an inverse relationship. Kidneys with more significant HN on average have less renal parenchyma; the SFU-HN grading system does have deficiencies that are especially evident in differentiation of severe HN (grades 3 and 4) [4,5]. Thus, the span between grades 3 and 4 is too large. Because renal parenchyma loss is a long gradual pathologic process, some kidneys with moderate HN (grades 2 or 3) demonstrate the onset of renal parenchyma thinning. We concur with the Onen US-HN grading system; specifically, the definitions of the SFU grades 3 and 4 were modified and mild or moderate renal parenchyma loss was added to the definition of grade 3. In the present work, a new grading system for UUTD, including HN and UD by MRU, was described and correlated with the SFU-US grading system.

To date, MRI remains an attractive imaging modality because of the absence of radiation exposure and superior resolution when compared to US. The MRU is easy to use and understand by a clinical urologist and can objectively show all images of the kidney and ureters at same time from different panels. The new MRU-UUTD grading system described the MUR image findings of each grade from different panels in detail, and correlated well with US-HN grade from the SFU. The difference between the systems at lower grades (grades 0–2) was minor, but markedly different at higher grades (grades 3 and 4). This new system also emphasizes the long process of gradual thinning of the renal parenchyma, narrows the span between grade 3 and 4, and seems more reasonable. The SFU grading system based on US lacks clarity and depends heavily on individual interpretation from the US examiner. The value of the MRU-UUTD grading system is that the subjective factor of visual interpretation is reduced for one observer over time, as are discrepancies between multiple observers. In its place, a more objective interpretation for UUTD is produced.

**Figure 7:**
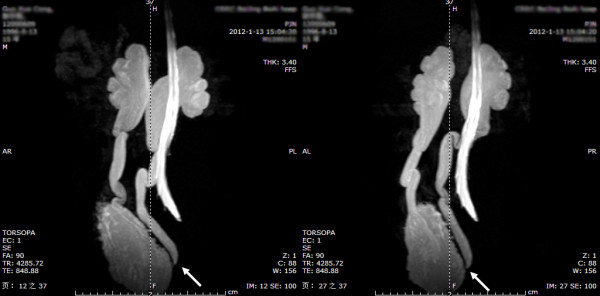
Ureteral stricture within the bladder wall and distal ureteral obstruction: HN and UD can be displayed in the same image of maximum intensity projection MRU during a 360-degree rotation, and vesicoureteral junction stricture or obstruction can be clearly shown (arrows: left and right ureters).

**Figure 8:**
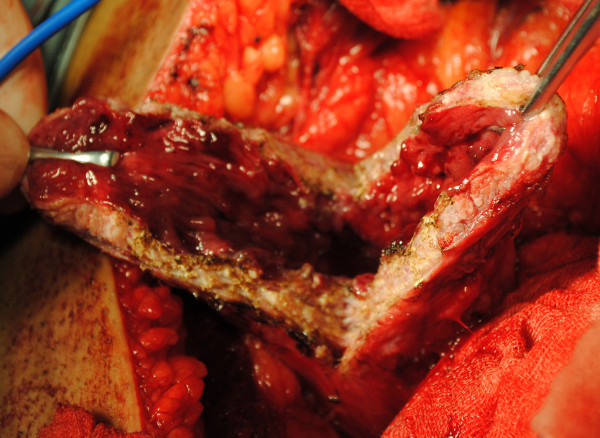
Fibrosis and thickening detrusor in a neurogenic bladder patient: fibrosis, thickening detrusor, and decreased bladder compliance secondary to progressive destruction of the bladder wall often result in ureteral stricture within the bladder wall and UUTD.

Another advantage of the MRU-UUTD grading system is that HN and UD can be observed comprehensively and described from the coronal and transverse panel MRU and the maximum intensity projection MRU. The HN and UD can be displayed in the same image of the maximum intensity projection MRU during a 360-degree rotation, and the vesicoureteral junction stricture or obstruction can be seen clearly (Figure 7). For patients with NB, the morphology and innervation of the ureterovesical junction play an important role in the incidence of VUR and UD, especially in patients with a long disease history. NB of long duration and irregular bladder management may account for the high percentages of HN, UD, VUR, and UUTD or UUT deterioration. The majority of our patients had moderate-to-severe HN and UUTD or UUT deterioration at the time of the initial evaluation. For these patients, detrusor fibrosis, detrusor thickening (Figure 8), and decreased bladder compliance secondary to progressive destruction of the bladder wall often result in ureteral stricture within the bladder wall and distal ureteral obstraction, which is an important factor in the etiology of UUTD, including HN and UD. The UUTD often results in chronic renal failure in patients with NBD. Therefore, pre-operative evaluation using this new MRU-UUTD grading system is important in the management of NB patients and can facilitate better informed clinical decision-making. Accordingly, the results of the UUTD management may improve as well. For example, earlier correction of UUTD has been shown to give better drainage from indwelling transurethral catheters. If the UUTD grade can be improved and the bladder condition is still poor, then AE can be performed. The renal parenchyma thickness is a predictor of recoverability of renal function [10]. Therefore, worsening HN and UD constitute a relative indication for AE surgery. However, some UUTD grades are an indication for AE, but other UUTD grades are not; grade 4 MRU-UUTD is not a good indication for AE due to excessive renal parenchyma loss. The new grading system can better discriminate grade changes in UUTD, provide objective indicators for UUT function, and is an important component of long-term follow-up of conservative and surgical treatment (Figure 6). The long-term outcomes of protection for UUT function by AE surgery using this new UUTD grading system will be the subject of another study. An examination of MRU is not expensive. The patients with HN, UD, tortuous knotting and vesicoureteral junction stricture really need a MRU instead of a simple renal US. In case only US would be done, the ureteral information especially in the ureteral obstruction due to the ureteral tortuous knotting and vesicoureteral junction stricture could be missing, and the patient could not be undergone the ureteral re-implantation during AE and the renal function could not be protected well.

## Conclusions

The new UUTD grading system is developed and described in detail based on the MRU findings. The new UUTD grading system correlates well with, and improves upon, the SFU-US grading system for HN. Because the new UUTD grading system provides an objective and comprehensive evaluation for HN and UD by MRU, the new UUTD grading system can be used to grade the UUTD and for longitudinal monitoring of UUTD, including pre- and post-operative observation. The new UUTD grading system emphasizes and subdivides the renal parenchymal status; this new system allows for better informed clinical decision-making, identifying changes in the UUTD and using follow-up for the outcome of treatment.

## Abbreviations

UUTD: Upper urinary tract dilation; HN: Hydronephrosis; UD: Ureteral dilation; MRU: Magnetic resonance urography; SFU: Society for fetal urology; NB: Neurogenic bladder; UUT: Upper urinary tract; US: Ultrasonography; AE: Augmentation enterocystoplasty; VUR: Vesicoureteral reflux.

## Competing interests

The authors declare that they have no competing interests.

## Authors’ contributions

LL have made contributions to conception, design, acquisition of data, analysis interpretation of data, drafting the manuscript. FZ helped to draft the manuscript. GC performed the statistical analysis. All authors read and approved the final manuscript.

## Authors’ information

LL, MD & PhD, is chairman of the department of urology of China Rehabilitation Research Center (CRRC), and a professor of urology and vice-chairman of urologic department of Capital Medical University in Beijing. His main interests are neurourology, urodynamics and incontinence. He is a committee member of the neurourology promotion committee of the international continence society (ICS), and was chairman of 42^nd^ ICS annual meeting in Beijing. Both FZ and GC are MD & PhD and urologist in the department of urology of the CRRC.

## Pre-publication history

The pre-publication history for this paper can be accessed here:

http://www.biomedcentral.com/1471-2490/14/38/prepub
